# Enhancing Psychiatric Competence: A Simulation-Based Approach to Improving Medical Students’ Knowledge and Confidence

**DOI:** 10.1192/bjo.2025.10435

**Published:** 2025-06-20

**Authors:** Iqra Saani

**Affiliations:** Pennine Care NHS Trust, Manchester, United Kingdom

## Abstract

**Aims:** Psychiatry is often considered a challenging specialty by medical students, largely due to the stigma attached to it. It also demands a unique skill set and relies heavily on interpreting subjective experiences, which can be a daunting task. Many students report hesitancy when approaching psychiatric patients, which indicates a need to bridge the gap between theoretical and practical learning. We believe that simulation is an effective way to achieve this. The aim of this Quality Improvement Project (QIP) was to assess and enhance medical students’ knowledge and understanding of common psychiatric conditions and instil confidence in them regarding psychiatric evaluation.

**Methods:** A hands-on simulation exercise was conducted on 04/07/24. Resident doctors, currently working in psychiatry, volunteered as simulators for the sessions. The scenarios included common psychiatric conditions such as depression, bipolar disorder, psychosis, anxiety disorder, schizophrenia, post-traumatic stress disorder, etc. There were a total of 8 stations, each comprising 15 minutes of history-taking, followed by 7 minutes for feedback and discussion with the simulator. Students also completed a questionnaire before and after the simulation, which assessed their understanding and confidence in handling psychiatric scenarios with a focus on history taking and risk assessment.

**Results:** The simulation was successfully conducted with all students participating actively. Pre- and post-simulation questionnaires revealed significant improvement in students’ understanding and confidence in handling common psychiatric scenarios.

Before the simulation, 38% of students reported feeling confident in conducting psychiatric history-taking and risk assessments. Afterwards, this figure increased to 85%. Furthermore, the percentage of students reporting good understanding of common psychiatric conditions increased from 44% to 87% after the stimulation.

An open-ended question revealed further support for these findings, with students expressing that the simulation helped them feel more comfortable approaching psychiatric patients and conducting interviews. A particular point noted by many students was the opportunity to receive immediate feedback from the simulator, allowing a clear explanation tailored to each scenario and the student’s performance/skills.

**Conclusion:** The Quality Improvement Project significantly improved medical students’ understanding and confidence in assessing common psychiatric conditions. Students reported increased comfort with history-taking and risk assessments, and specifically commended the value of realistic scenarios and immediate feedback. Based on these results, we aim to continue this initiative for the next cohort of students and integrate it as a regular component of the psychiatric education programme at the Irwell Unit, Pennine Care NHS Trust.

